# 19 Minced Skin Grafts Can Be Expanded up to 500 Times to Re-epithelialize Full-thickness Burns

**DOI:** 10.1093/jbcr/irac012.023

**Published:** 2022-03-23

**Authors:** Kristo Nuutila, David E Varon, Michael Broomhead, Elof Eriksson

**Affiliations:** U.S. Army Institute of Surgical Research, San Antonio, Texas; University of Michigan Medical School, San Antonio, Texas; Applied Tissue Technologies LLC, Hingham, Massachusetts; Applied Tissue Technologies LLC, Hingham, Massachusetts

## Abstract

**Introduction:**

Deep and large burns require hospitalization and, without exception, surgical treatment. Skin graft expansion in major burns currently is restricted to a 1:6 expansion ratio (possibly 1:9) by current methods. For this reason, skin transplants are limited by donor site availability for patients with extensive burns, who may have only small areas of healthy skin for use as donor sites. The objective of this project is to demonstrate the efficacy of pixel grafting, to produce split-thickness skin grafts (STSG) and dermal grafts which can be expanded at least 100 times (1 cm^2^ donor skin will cover 100 cm^2^). Pixel grafts are skin grafts that are prepared from split-thickness donor skin that is 0.3 mm (12/1000 of an inch) thick and minced into pieces that are 0.3 x 0.3 mm. The methodology relies on mincing a small area of donor skin using a hand-held mincer to create uniform skin pieces, each of which will serve as a small center of skin regeneration.

**Methods:**

Deep partial-thickness burns were created on the dorsum of Yorkshire pigs. Three days post injury the burns were debrided and grafted with epidermal/dermal and dermal pixel grafts using expansion ratios from 1:2 to 1:500. The pixel grafts were prepared by harvesting 0.3 mm thick skin grafts from the same donor site going down in depth (Layer 1: STSG; Layer 2: dermal graft). Subsequently, the grafts were minced to 0.3 x 0.3 x 0.3 mm pixel grafts, suspended in a small volume of hydrogel and transplanted onto debrided porcine burn wounds. Healing was monitored up to 28 days and biopsies were collected on days 6, 10 and 14. The wounds were excised and fixed in formalin for histologic analysis. In addition, to measure wound area reduction and wound depth, wound images were taken. Multiple wound healing parameters were used to assess the quality of healing and the results were compared to STSG (standard of care treatment).

**Results:**

Histology demonstrated that when both STSG pixel grafts and dermal grafts were transplanted onto porcine full-thickness wounds in a 1:2 ratio they were able to fully re-epithelialize the wounds in 14 days. At day 14, re-epithelialization-% using other expansion ratios varied from 46 % (1:500) to 64 % (1:10) and by day 28 all the wounds were fully re-epithelialized. Wound images obtained at different time points showed that by day 28 all the pixel graft grafted wounds had reduced in area efficiently, from 70 % (dermal pixel grafts 1:50) to 81% (STSG 1:100). No statistically significant differences were observed between the pixel graft study groups and STSG (standard of care).

**Conclusions:**

This study concluded that both epidermal/dermal and dermal pixel grafts can be suspended in a hydrogel and transplanted onto wounds regardless of orientation (dermal side up or down), making the transplantation greatly simplified.

